# Supplementary material for: Weathering the storm of COVID-19 pandemic: A cross-sectional survey of reported changes in first contact physiotherapy services in the UK and Australia
Source: PLoS One. 2026 Jan 21;21(1):e0340995. doi: 10.1371/journal.pone.0340995 (PMC12822978; doi:10.1371/journal.pone.0340995)
Supplement: S1 Checklist — (DOCX) [file pone.0340995.s001.docx]

## **S1 Checklist. Checklist for Reporting Results of Internet E-Surveys (CHERRIES) for FCPS survey**

| **Item category** | **Check-list item** | **Explanation** |
| --- | --- | --- |
| **Design** | Survey design | International cross-sectional survey of first contact physiotherapists for musculoskeletal patients in the UK and Australia (n=211). |
| **IRB (Institutional Review Board) approval and informed consent process** | Ethical approval | A favourable ethical opinion was obtained from relevant authorities. |
|  | Informed consent | The initial page of the survey introduced the study to participants by providing detailed information about the study through an embedded participant’s information sheet and a consent form. The consent was set up as a requirement for participants to proceed with the survey |
|  | Data protection | Data was collected with Qualtrics, a secure platform for online surveys. All data was downloaded to Excel and deleted from Qualtrics at the close of the survey. All answers were anonymous except those that provided their contact details for further study. Personal contact details were stored in a sperate file from the anonymised data using a secured server located in both countries. |
| **Development and pre-testing** | Piloting | Several parts of the survey include validated frameworks such WHO responsiveness framework and the FRAME, however, content and face validity, including feasibility and time required were assessed. To assess content and face validity, survey was pilot tested with 12 Physiotherapists in FCPS role in the UK and Australia using a Qualtrics link, employing a 1–5-point scale and encouraging open comments. Eight responses were received. In addition, feasibility and time required to complete the survey was assessed, the completion time averaged 13.9 minutes (SD 10.0), with a 100% unit response rate. Participants found the questionnaire content relevant to FCPS and the research question. Some minor terminology adjustments and clarification of the survey's time frame were suggested. To address time frame concerns, two participants were consulted by telephone, highlighting inconsistencies like "when the change first occurred," "during COVID," and "due to COVID." These issues were subsequently resolved, and adjustments were made to enhance question clarity and structure.  In addition, the reliability of the survey instrument was conducted using Cronbach’s Alpha. (Please see details in the manuscript) |
| **Recruitment process and description of the sample having access to the survey** | Open survey | The survey was accessible to all first contact physiotherapists in the UK and Australia who encountered the study advertisement and survey link via professional organisations, closed professional groups, or various social media platforms like Facebook, Twitter, and LinkedIn. |
| **Survey administration** | Contact mode and advertising the survey | Qualtrics link with study information was posted on the interactive Chartered Society of Physiotherapy (CSP) online forum [interactiveCSP \| The Chartered Society of Physiotherapy](https://www.csp.org.uk/icsp). The website was created by the CSP for the rollout and discussion around in the UK. Furthermore, the Qualtrics link with study information was sent to the Australian Physiotherapy Association for dissemination to physiotherapists in FCPS roles in Australia. The study was further disseminated on the web using first contact physiotherapy Facebook groups, twitter, and LinkedIn. |
| **Survey administration** | Web-based link | The survey was hosted by Qualtrics which automatically captured responses. |
|  | Voluntary/involuntary | The survey was voluntary. No incentives were offered. |
|  | Timeframe | The survey ran from January to April 2023. An average of two reminders were sent out across all platforms. |
|  | Randomization of items | Survey items were not randomised. |
|  | Adaptive questioning | Some items displayed conditionally based on responses to earlier items. Also, skip function was used in 10 questions, therefore, certain items only displayed conditionally based on responses to earlier items |
|  | Number of questions/items | The survey contained a total of 53 questions and took an average of 15 minutes to complete. |
|  | Number of pages | The total number of webpages was 40.  Due to the skip patterns, the total number  of pages a given respondent would see would be less than this. |
|  | Completeness check | Completing all questions was not mandatory. The respondent could submit the survey without fully completing the survey. |
|  | Review check | Respondents were able to review and change the answers with a “back” button. |
| **Preventing multiple entries from the same individual** | IP-check, | IP addresses were employed to detect and review multiple data entries originating from the same individual or location and 13 duplicates were identified. Among the 13 duplicates identified through IP addresses, 12 exhibited distinct responses, including variations in age, gender, and years of experience. In cases where participants provided their email addresses, these email addresses were also dissimilar, indicating that different individuals used the same office computer to complete the survey. However, one entry, lacking sufficient data for verification, was removed from the analysis |
| **Analysis** | Handling of incomplete questionnaires | All responses were analysed, and all incomplete item and “I do not know” or “not applicable” response were considered missing data and handled using pairwise deletion. |
|  | Timestamp | No specific time limit was set for completing the survey. Participants were permitted to revisit and complete their surveys at their convenience throughout the data collection period. |
|  | Statistical correction | No correctional analysis was made. |
