# Supplementary material for: Weathering the storm of COVID-19 pandemic: A cross-sectional survey of reported changes in first contact physiotherapy services in the UK and Australia
Source: PLoS One. 2026 Jan 21;21(1):e0340995. doi: 10.1371/journal.pone.0340995 (PMC12822978; doi:10.1371/journal.pone.0340995)
Supplement: Attachment — Submitted filename: Responses to reviewers and editor.docx [file pone.0340995.s005.docx]

**Responses to reviewers and editor**

*We sincerely appreciate the editor and reviewers for their time, commitment, and effort in helping to strengthen our paper. We have addressed the concerns raised below. The main document is marked with tracked changes. Also, a clean copy has been attached.*

**REVIEWER 1**

I generally found that the conclusion sections in both the abstract and main text did not accurately reflect the findings of the analysis outlined in the Results and Discussion section, and at times included statements that appear to be unsubstantiated by the study. I would suggest that these sections are revised. Please find my specific comments below.

1. Weathering the storm of COVID-19 pandemic in UK and Australia First Contact Physiotherapy
Services: A cross-contextual survey investigating the timing and type of rapidly implemented
changes
Could the authors please clarify how they defined “rapidly implemented changes”, and in what way the questionnaire ensured that respondents only focused on these? Note that although one of the main findings of the survey is that the timing of changes implementation was mostly rapid (within 3 months), this does not mean that the survey was investigating rapidly implemented changes.

***Response:*** *The title has been revised for consistency and to more accurately reflect the study’s focus. The term "rapidly implemented" was originally intended to capture the speed at which FCPS responded to the pandemic. We used the definitions from a recent systematic review (Adeniji et al., 2025) that reported that most healthcare service adaptations during the COVID-19 pandemic were implemented within the first three months following the WHO’s declaration that would be considered a rapid response during a crisis. Although the term "rapidly implemented" has been removed from the title and throughout the paper for consistency, this study still uses the timeline identified in the literature as a reference point for categorising the timing of changes. We ensured that the respondents only focused on the initial changes by including questions related to the timing of the changes in the questionnaire (<3 months and 3-6 months, etc).*

2. Aim: This study aims to explore the type, and the timing of changes implemented in FCPS during the COVID-19 pandemic in the UK and Australia. The manuscript frequently references "changes implemented" or "adaptations" in service provision (including in the title). I have two concerns in this regard: - The changes considered in the survey (Qs 10 to 26) do not differentiate between those formally introduced as policy changes by healthcare providers and unintended changes that may have arisen due to other factors. Such factors include shifts in population needs (e.g., linked to lifestyle changes associated with lockdowns), alterations in healthcare-seeking behaviours, or modifications in healthcare providers’ working patterns that were demand- rather than policy-driven. The authors should clarify throughout the manuscript that the study aimed to document perceived changes in service provision, without implying that these changes were necessarily the result of “adaptations” by healthcare providers. In this particular sentence, I would also clarify that these changes were “self-reported by FCPS providers”.- The study only considered *initial* changes to FCPS (see point 5). This should be clarified throughout the manuscript.
***Response:*** *Thank you for making this distinction. We have now revised the paper throughout to	talk about “initial”, “self-reported” and “perceived” changes. This has also been established in the	introduction as below:*

*“Therefore, this study aims to explore changes in FCPS during the COVID-19 pandemic in the	UK and Australia, with a focus on the initial changes. This approach helps to gauge the speed	of FCPS response and readiness based on physiotherapists’ self-reported experiences in the	FCPS role”.*

3.From the questionnaire “For some, changes occurred in the first three months of the COVID 19 pandemic while it took more months for others.” One of the main findings of this study appears to be that the initial changes to MSK service delivery were mostly implemented during the first three months of the global pandemic. I therefore find it concerning that the introduction to the questionnaire appears to be highlighting “in the first three months” as a plausible answer to the timing questions Qs 5 and 7. Although “more months” is mentioned in the same sentence, it aggregates all other possible answers to these questions into one category, potentially emphasising the first timeframe more strongly. This is concerning given that respondents are being asked to recall events that occurred three years prior, making their recollections susceptible to suggestion. How do the authors justify the need to include this sentence in the questionnaire’s introduction? This potential source of bias should be acknowledged in the discussion.

***Response:*** *The survey used event timelines to prompt respondents' memories, to minimise recall bias, helping participants to contextualise their perspectives and experiences within a specific timeframe (Moreno-Serra et al., 2022). We acknowledge that using a time prompt may introduce potential leading, resulting in bias, this has now been addressed in the limitations section.*

4. From the questionnaire “The following set of questions will explore changes to access, mode of delivery, condition managed, interventions provided, patient case load, and administrative duties in your FCP service. The responses you provide should be based on the first changes that occurred in your FCP service delivery for MSK patients during the pandemic” Participants were asked to describe the “first changes” that occurred in service delivery. This phrasing is ambiguous, as it is unclear:- Whether these changes should be interpreted as formal policy implementations or simply as observations of shifts in practice (e.g., changes in healthcare-seeking behaviour);- Which period of time respondents should have considered as representing the “first changes.” Does this refer to any period until another change was introduced (or observed) in service provision? How significant must a subsequent change have been to delineate the timeframe? - How it relates to previous questions on service suspension; Given these ambiguities, how confident are the authors that participants would have interpreted these instructions in a consistent manner? And how certain are they that respondents could accurately recall time variations in service provision three years later, particularly considering the likelihood of multiple policy changes throughout the pandemic on timescales of weeks/months?

***Response:*** *Thank you for this thoughtful observation. We acknowledge that the phrasing regarding the "first changes" could have introduced variability in interpretation among respondents. To address these concerns:*

*Formal vs. observed changes: Given the rapid and dynamic nature of the pandemic response, we intentionally kept the definition broad to reflect the real-world experiences of clinicians, recognising that formal policies were sometimes preceded by formal/informal changes at the service level, especially when considering a significant event such as the COVID-19 pandemic.*

*Timeframe and subsequent changes: Participants were prompted to focus on the earliest significant change they experienced in service delivery, rather than later developments. While we recognise that subsequent changes could have blurred the memory of initial changes, the survey specifically asked about the first discernible changes to mitigate this issue.*

*Relationship to service suspension questions: Where questions about service suspension were asked separately, they aimed to determine if services ceased altogether, while the "first changes" questions referred to modifications in how services were delivered (e.g., move to telehealth, triage adjustments).*

*Consistency and recall accuracy: We acknowledge the potential for variability and recall bias, particularly given the time elapsed since the onset of the pandemic. This was noted as a limitation in our study. However, to help mitigate this, we used prominent public events (e.g., WHO's pandemic declaration) as temporal anchors and included timeline prompts within the survey to aid recall and provide a consistent reference point across participants. In addition, the survey was designed so that the selected timeframe was consistently carried through all questions, helping to prevent confusion or mixing of timelines.*

*While there is inevitably some uncertainty around consistency of interpretation and memory, we believe the methods employed were reasonable given the retrospective nature of the study and the aim to capture real-world practice changes during an unprecedented period. In addition, even though we have anchored the development of the survey on the latest evidence and best practice, we acknowledge that recall bias, variable definitions and the potential for different interpretations are inherent in survey research. These limitations have been acknowledged.*

5. This study aims to explore the type and the timing. I have two concerns regarding the use of the term “timing” in this context. Firstly, the study focuses exclusively on the initial implementation of changes to service provision and does not account for how these changes may have evolved throughout the pandemic. This limitation means the study cannot determine whether more significant changes occurred at later stages of the pandemic, or how long those initial changes lasted (see point 4). Since the phrase “timing of changes” typically implies not only their onset but also their progression over time, its use here may be misleading. I suggest making it clear that the study concerns only the onset of changes.

***Response:*** *The title has been rephrased to accurately reflect the scope of the study. We acknowledge the importance of assessing longitudinal changes throughout the course of the pandemic, this is out of scope of this project. The study aim has been revised to clarify the focus on the initial changes that may have been introduced in practice. The revision helps eliminate any misleading interpretation and better captures the study’s real intent. In addition, the paper has been revised throughout to more accurately reflect the findings, particularly in clarifying the initial onset of changes as perceived by providers, where applicable.*

Secondly, I am concerned about the use of a “universal” time frame to calibrate the timing of changes. As noted in the manuscript, “Each country responded differently to the COVID-19 pandemic,” and “lockdown measures were implemented across Australian states with varied timing.” Furthermore, the timing and severity of pandemic waves differed significantly between countries, regions, and states. How do the authors justify using the declaration of COVID-19 as a global pandemic (by whom? Please add) as a uniform reference point across the UK and Australia, considering these contextual differences?

***Response:*** *Thank you for this thoughtful observation. We fully acknowledge the variability in the timing and severity of COVID-19 waves and responses across countries, regions, and even states. However, the decision to use the World Health Organisation’s (WHO) declaration of COVID-19 as a global pandemic (March 11, 2020) as a reference point was based on its symbolic and practical value as a widely recognised turning point in public and institutional awareness. This declaration marked a critical shift in global and national responses, often triggering more formalised actions, including lockdowns, resource reallocation, and shifts in healthcare delivery. Although the implementation of these measures varied, insights from our preliminary one-to-one consultations during the study's conceptualisation phase, along with existing literature, indicated that the WHO declaration served as a common temporal anchor in both professional and public consciousness. Using this reference point also enhances comparability across diverse settings, particularly in an international study where more context-specific time frames may complicate analysis and interpretation. We have now clarified this rationale (under methods) and added who made the declaration in the revised manuscript. The contextual differences between countries and settings under study are acknowledged in the introduction.*

6. “An international cross-sectional study was conducted, using an online survey tool. Data were
collected from January-April 2023 from physiotherapists managing MSK patients in PC and ED across the UK and Australia, respectively”. It should be made clear that the data was collected from physiotherapists who fulfilled the study’s eligibility criteria, which stated that only responses from providers who recalled changes in MSK service delivery would be considered.

***Response****: Information on eligibility has now been added to the abstract (methods) and manuscript sampling section as shown in query no 13.*

7. One hundred and fifty-three participants were included in the analysis with 64.1% based in the UK and 35.9% in Australia. This should be in the methodology, not the results.

***Response:*** *This has now been removed from the abstract results and moved to the abstract methods section as shown in query no 13.*

8. The main age group were 35-44 years (42.2%), with 59.3% females. The age and gender of the provider was not shown to be significant in any part of the analysis. Why is it included as a main result in the abstract?

***Response:*** *Thank you for your comment. This has now been removed as shown in query no 13.*

9. 75.7% of the changes in the delivery…The questionnaire only asked participants when the first changes took place, so this sentence should read "75.7% of initial changes" or similar.

***Response:*** *This has now been revised to “initial changes” as shown in query no 13*

10. “75.7% of the changes in the delivery of FCPS mostly occurred during the initial three months
of COVID-19’s declaration as a global pandemic” What does “mostly” mean in this sentence?

***Response:*** *This was a typo error, and “mostly” has now been removed as shown in query no 13*

11. The timing and country had a mixed effect on the variables of change.
Please report the most significant results in the abstract.

***Response:*** *The abstract has been re-written and significant results have now been added as shown in	query no 13.*
12. Adaptations in FCPS during the COVID-19 pandemic varied across countries. This study only considered two countries and therefore does not provide enough statistical evidence to support this statement. Furthermore, due to the complete overlap between the country and the type of service delivery setting considered (PC settings in the UK and ED settings in Australia), it is impossible to determine whether similar delivery settings in the two countries experienced different or similar changes in FCPS provision. To substantiate such a claim, the authors would need to collect additional data from PC settings in Australia and ED settings in the UK and account for. these variables in their analysis. With regards to the use of the term “adaptations” in this context, please refer to comment 2.

***Response:*** *The term "adaptation" has been replaced with "changes" in the abstract and throughout the	study to ensure consistency and clarity as shown in query no 13. The abstract has now been re-written,	and we revised the paper throughout to reflect that only two countries were included and that due to	the nature of the FCPS in the UK and Australia the settings were different as shown in query no 13.*

13. This variation underscores the need for timely and context-specific improvement strategies
Variation in observed changes between two different countries in itself doesn’t underscore a) the need for improvements, or b) the need for these improvements to be “context specific” (observing variation does not rule out the possibility that adaptations introduced in country A would have led to better outcomes in country B had they been applied there too). Furthermore, the analysis of the survey data did not investigate the link between the type of changes observed and the quality of service delivered and therefore cannot provide any conclusions as to what type of changes - context specific or not - led to better outcomes. Please rewrite the conclusions to better reflect the findings reported in the results section.

***Response:*** *The whole abstract has been re-written to better reflect study and findings as below:*

*“During the COVID-19 pandemic, First Contact Physiotherapy Services (FCPS) in UK primary care (PC) and Australian emergency departments (ED) initially faced disruptions in managing musculoskeletal (MSK) patients, leading to rapid changes in service delivery. However, there is limited understanding of these changes, especially those that occurred during the early phase of the pandemic, which could improve our knowledge of FCPS responsiveness and readiness for future crises. This study explores the initial changes in FCPS during the COVID-19 pandemic in the UK and Australia. A cross-sectional survey was conducted between January and April 2023. Data were self-reported by physiotherapists managing MSK patients in UK PC and Australian ED. Only responses from those who recalled changes in FCPS were included, with 153 participants analysed. Descriptive statistics and two-way ANOVA examined the effects of timing of change, country, and their interaction on readiness and response to patient needs. Overall, 75.7% of initial changes were perceived to have occurred within three months following the World Health Organisation’s declaration of COVID-19 as a global pandemic. Participants from both countries differed significantly in their perceptions of how COVID-19 affected patient access to FCPS (p < 0.001). Changes in MSK patient presentation to FCPS varied significantly by both the timing of the change and the country (p < 0.001). Similarly, changes in the care delivery platform were significantly associated with the timing of change (p = 0.014) and the country (p < 0.001). While participants reported inadequate readiness and moderate responsiveness, there were no significant effects of timing or country found on these outcomes. This highlights the need to learn from past experiences, build on successful strategies, and strengthen FCPS to enhance their capacity and resources to meet demand. Doing so will improve their readiness for future crises, minimise service disruptions, and support the resilience of FCPS”.*

14. The UK reported 59.2% (n=42) increased use of the triaging process
In this and many other instances where results are reported (e.g. In terms of intervention provided during the pandemic, self-management increased more in the UK 86.8% (n=59), compared to Australia 44.7%) the imprecise phrasing makes the meaning of the sentence open for misinterpretation: As is, the sentence may be incorrectly interpreted as suggesting that the UK reported a 59.2% increase in the triaging process, whereas it should read “Among UK-based respondents, 59.2% observed an increased use of the triaging process”. Please correct this throughout the manuscript, and also in the Tables.

***Response:*** *Findings have been rephrased where applicable, also, Table 3’s title has been revised and	table footnote/legend included to enhance correct interpretation of the table. Below is the included Table	footnote/legend:*

*This table presents observed initial changes in FCPS in terms of the level of use of different FCPS access route, platforms for delivering care, interventions provided, including the levels of patient presentation, and staff workload, as perceived by physiotherapists working in FCPS roles in UK primary care and Australian emergency departments.*

15. The COVID-19 pandemic led to changes in FCPS within the first three months of its declaration as a global pandemic in both the UK and Australia. The “eligibility criteria” used to select the 153 survey participants considered in this analysis means this sample cannot be used to determine whether any statistically significant changes to MSK service provision occurred during the COVID-19 pandemic in either country. This sentence, therefore, is unsubstantiated unless a reference is provided or further analysis conducted. The survey collected raw data (which was excluded from the sample of data analysed) from respondents who reported observing no changes in service provision during the pandemic (20.3% overall). If analysed, this data could provide some evidence in support of this statement. However, the title of the survey itself (“Exploring changes to first contact physiotherapy services for musculoskeletal patients during COVID-19 Pandemic”) and stated objectives is very likely to have introduced a bias in the collection of responders, favouring participation from individuals who indeed observed changes in service provision. The authors should either a) conduct an analysis on the statistical significance of the claim that the COVID19 pandemic led to changes in FCPS from their complete raw data sample while providing a satisfactory explanation for why the above-mentioned bias is not a cause for concern, b) provide a reference for this sentence, or c) remove this sentence.

***Response:*** *We acknowledge the reviewer’s concern and agree that, due to the eligibility criteria applied, the analysed sample may not be representative for establishing statistical significance regarding changes in MSK service provision during the COVID-19 pandemic. The sentence in question has been revised to avoid implying a conclusive statement about FCPS. We have removed the statement in question and revised to a more qualified statement. Queries no 15-18 focused on the conclusion section and the whole conclusion has been revised as below:*

*“Changes in FCPS were reported to have commenced within the first three months following the World Health Organisation’s declaration of the COVID-19 pandemic, in both the UK and Australia. These changes likely varied due to differences in the contexts in which FCPS operate [10, 11], potentially influenced by local policies, capabilities, service needs, supply and demand during the COVID-19 pandemic [6, 15-18]. Even though changes were reported to have occurred within the first three months in both contexts, both demonstrated similar levels of inadequate readiness for rapid changes in service delivery and moderate levels of responsiveness to the healthcare needs of MSK patients. This underscores the need to learn from past experiences, invest in the wins and support FCPS to build resilience in their operations to rapidly assess demand, match supply and create flexible and agile plans to prevent or minimise service disruptions in future crises”.*

16. These changes varied depending on contextual factors influenced by policies, needs, supply and demands during the pandemic. This study did not consider the role of policy implementation, nor collect statistics on the needs in the local population, or on the “demand” during the pandemic. This statement therefore is not substantiated by the study.

***Response:*** *The sentence has been revised to avoid implying a conclusive statement about FCPS. We have revised to a qualifying statement supported with references as shown in query no 15.*

17. For instance, patients continued to have access to the FCPS due to the availability of telehealth in the PC setting, while the ED saw a significant reduction in MSK patient presentations, as it primarily continued with traditional face-to-face services based on the need for hands-on acute care.
This statement seems to be in direct contradiction with the earlier statement that “Among UK respondents, 60.0% (n=53) reported suspensions, compared to only 9.3% (n=5) of respondents from Australia”. How do you reconcile the two statements?

***Response:*** *This was an error, and it has been removed. The whole discussion has been revised as	shown in query no 15*

18. This underscores the necessity for the development of context tailored implementation
Since the relationship between observed changes (some of which may have been beyond the control of providers and therefore fall beyond the scope of ‘implementation’), “context” (how is context defined?), and outcomes haven't been investigated in this analysis, this statement is unsubstantiated by this study.

***Response:*** *The sentence has been revised to accurately reflect findings as shown in query no 15.*

**REVIEWER 2**

This is an interesting topic and author write it well but still need some gramatical corrections. Valid stat analysis is done. authors has makrked all the valuable points in manuscript. Despite some corrections are required which are mention below:

1. introduction need to be deined more explaining about the topic

***Response:*** *The paper has been extensively revised to better reflect the scope to the study, including the	introduction*

2. discussion also need some minor corrections

***Response:*** *We have also revised the discussion as per the feedback from reviewers*

**EDITORIAL REQUEST**

1. Please ensure that your manuscript meets PLOS ONE's style requirements, including those for file naming. The PLOS ONE style templates can be found at

https://journals.plos.org/plosone/s/file?id=wjVg/PLOSOne_formatting_sample_main_body.pdf and

<https://journals.plos.org/plosone/s/file?id=ba62/PLOSOne_formatting_sample_title_authors_affiliations.pdf>.

***Response****: The manuscript has been revised in line with PLOS ONE requirements.*

2. Please provide additional details regarding participant consent. In the ethics statement in the Methods and online submission information, please ensure that you have specified what type you obtained (for instance, written or verbal, and if verbal, how it was documented and witnessed). If your study included minors, state whether you obtained consent from parents or guardians. If the need for consent was waived by the ethics committee, please include this information.

***Response:*** *Ethics information and participants consent has been provided under the methods section, and online submission information.*

3. We note that you have indicated that there are restrictions to data sharing for this study. For studies involving human research participant data or other sensitive data, we encourage authors to share de-identified or anonymized data. However, when data cannot be publicly shared for ethical reasons, we allow authors to make their data sets available upon request. For information on unacceptable data access restrictions, please see http://journals.plos.org/plosone/s/data-availability#loc-unacceptable-data-access-restrictions.

Before we proceed with your manuscript, please address the following prompts:

a) If there are ethical or legal restrictions on sharing a de-identified data set, please explain them in detail (e.g., data contain potentially identifying or sensitive patient information, data are owned by a third-party organization, etc.) and who has imposed them (e.g., a Research Ethics Committee or Institutional Review Board, etc.). Please also provide contact information for a data access committee, ethics committee, or other institutional body to which data requests may be sent.

b) If there are no restrictions, please upload the minimal anonymized data set necessary to replicate your study findings to a stable, public repository and provide us with the relevant URLs, DOIs, or accession numbers. Please see http://www.bmj.com/content/340/bmj.c181.long for guidelines on how to de-identify and prepare clinical data for publication. For a list of recommended repositories, please see https://journals.plos.org/plosone/s/recommended-repositories. You also have the option of uploading the data as Supporting Information files, but we would recommend depositing data directly to a data repository if possible.

Please update your Data Availability statement in the submission form accordingly.

***Response****: All relevant data are included within the manuscript. Raw data are available via the University of Surrey's open access repository. The DOI has been added.*

4. Please note that your Data Availability Statement is currently missing [the repository name and/or the DOI/accession number of each dataset OR a direct link to access each database]. If your manuscript is accepted for publication, you will be asked to provide these details on a very short timeline. We therefore suggest that you provide this information now, though we will not hold up the peer review process if you are unable.

***Response****: DOI provided.*

5. Please include a caption for figure 1.

***Response***: *Figure 1 captions included*

6. Please include captions for your Supporting Information files at the end of your manuscript, and update any in-text citations to match accordingly. Please see our Supporting Information guidelines for more information: http://journals.plos.org/plosone/s/supporting-information.

***Response:*** *Captions provided at the end of manuscript and updated in-text*
