# Supplementary material for: Weathering the storm of COVID-19 pandemic: A cross-sectional survey of reported changes in first contact physiotherapy services in the UK and Australia
Source: PLoS One. 2026 Jan 21;21(1):e0340995. doi: 10.1371/journal.pone.0340995 (PMC12822978; doi:10.1371/journal.pone.0340995)
Supplement: Attachment — Submitted filename: Response to reviewers .docx [file pone.0340995.s006.docx]

**Response to reviewers and editor**

We sincerely thank the reviewers and editor for their thoughtful and constructive feedback. The manuscript has been carefully revised in response to the comments, with all updated sections clearly highlighted in yellow for ease of reference. Detailed responses to each point raised are provided below.

**Reviewer #1:** The manuscript repeatedly states (in the abstract, results, and conclusions section) that “both [countries] demonstrated similar levels of inadequate readiness for rapid changes in service delivery and moderate levels of responsiveness to the healthcare needs of MSK patients”. I believe at present the analysis has not sufficiently demonstrated either claim. With regards to the claim of “moderate levels of responsiveness”: The study should more clearly define the use of the term “moderate”. In the text, however, this is discussed as being indicative of an issue with responsiveness (e.g. “The moderate level of FCPS responsiveness could be attributed to *inadequate* readiness”, “the changes reported in FCPS in the UK and Australia during the pandemic generally reflect the experiences of changes reported in healthcare service [..] exposed the fragility of the current health systems and their unpreparedness to deal with the turbulence of the unexpected”).
However, from what I can see from Fig. 1B, in the UK only in the case of “Choice (in person or virtual)” did a majority of respondents disagree with the statement “I believe my FCP services met the patient's expectations and needs”. In the case of Australia, in none of the criteria a majority of respondents disagreed with that statement. If only in a minority of cases were issues identified with the level of care received by the patients, why is this indicative of an issue with responsiveness? In what way does it reflect “the fragility of the current health systems and their unpreparedness to deal with the turbulence of the unexpected”? With regards to the claim of “inadequate readiness”: It is unclear how the authors determined that both settings demonstrated levels of inadequate readiness; in particular:
Based on Fig 1A, respondents were asked to express their opinion on readiness in 15 different areas. However the results section only reports answers to five (access to information, effective leadership, stakeholder buy-in, tech support, and infrastructure); why was only this subset considered? Why were areas as important as “Outputs & Results” not included? The criteria used to determine whether there is overall agreement with a particular statement are unclear. Based on my understanding, the survey asked respondents whether they “agreed,” “disagreed,” or were “neutral” regarding readiness in various areas. When reporting overall agreement with readiness, the authors appear to use a majority rule—that is, they consider respondents to be in agreement if more than 50% selected “agree”. However, it seems the authors apply a different standard when interpreting disagreement.
Specifically, they appear to treat disagreement with readiness as equivalent to agreement with a lack of readiness (this in itself should be explicitly stated in the text as an assumption), but without requiring a majority for overall interpretation. For example, in support of the statement that “participants agreed there was a lack of readiness for change in terms of infrastructure,” they cite agreement levels of 42.4% (UK) and 39.0% (Australia)—both below the 50% threshold. By contrast, in other areas such as access to information or effective leadership, the authors emphasize whether a majority agreed, suggesting inconsistency in their criteria for interpreting overall agreement levels. I believe their reasoning is that, in the case of the UK, more respondents selected “disagree” than selected “agree” or “neutral” individually. However, this still means that only a minority (<50%) of participants explicitly disagreed with the statement of readiness. Therefore, I do not think the authors can reasonably claim that respondents “agreed that there was lack of readiness for change in terms of infrastructure” based on this result. In the case of Australia, however, I believe the authors have made a mistake when quoting 39% in disagreement with readiness of infrastructure, based on Fig 1A. Please clarify the discrepancy. Based on the subset of 5 areas considered, it would appear that only in 2 out of 5 cases a majority of respondents thought that their FCPS was not ready for rapidly implemented changes. In what way does this support the statement of “inadequate readiness for rapid changes in services”? To establish whether this claim can be supported, authors should 1) use consistent criteria of majority, and 2) summarise all areas investigated in the survey (especially “results and outcomes”) or motivate why only 5/15 are relevant. Overall, I believe the survey results are more nuanced—and at times contradictory to existing literature—than currently acknowledged. The conclusions should reflect this complexity, not just for the sake of accuracy, but also because it adds to the interest and value of the study.

***Response:*** *We thank the reviewer for this detailed insight.* The paper has now been revised to apply consistent criteria of agreement for determining the majority, using an arbitrary cut-off of ≥50% to indicate moderate to high agreement and <50% for low agreement. The cut-off value has also been included under the data analysis section (page 7). *We acknowledge that the reported 39% disagreement regarding infrastructure readiness was an error, and it has been cancelled. All readiness and responsiveness result items have been presented accordingly. The abstract, discussion (pages 19 and 20* ***on the manuscript with track changes****) and conclusion have also been revised accordingly.*

The results have been revised as shown on pages 13 and 14 and as below:

**Readiness and responsiveness with changes**

*“In general, strong agreement was consistently reported for the readiness factors of access to information and leadership, with stakeholder’s buy-in recording no agreement across both the UK and Australia. In the UK, participants reported moderate to high agreement (≥50%) with the following four readiness items, presented in descending order of agreement: access to information (72.7%, n = 48), leadership (65.5%, n = 42), mission and vision (55.2%, n = 32), and output and results (51.7%, n = 30). In contrast, the remaining items showed low levels of agreement (<50%), including: compatibility (48.5%, n = 32), infrastructure (39.4%, n = 26), external partnerships and support (39.3%, n = 22), financial resources (38.1%, n = 16), learning strategy (35.7%, n = 20), supplies and procurement (35.5%, n = 32), availability of emergency response guide (35.5%, n = 22), human resources (37.9%, n = 22), human resources management (20.7%, n = 12), technical support (6.1%, n = 4), and stakeholders’ buy-in (0%). In Australia, moderate to high agreement (≥50%) was also reported for three readiness items, listed in descending order: supplies and procurement (73.7%, n = 28), access to information (63.4%, n = 26), and leadership (53.7%, n = 22). All other items recorded lower levels of agreement (<50%), including availability of emergency response guide (48.7%, n = 19), financial resources (43.2%, n = 16), mission and vision (41.0%, n=16), human resources management (46.2%, n=18), learning strategy (40.5%, n=15), human resources (39.0%, n=16), compatibility (32.4%, n=12), external partnership and support (24.2%, n=8), output and results (19.5%, n=8), infrastructure (14.6%, n=6), technical support (9.5%, n=4), and stakeholders’ buy-in (0%).*

*Regarding responsiveness, communication, autonomy, equitability and prompt attention (timeliness of care) demonstrated notable levels of agreement across both the UK and Australia.* ***Participants from the UK reported moderate to high agreement (≥50%) with seven responsiveness factors, listed in descending order: communication (81.3%, n = 52), autonomy (65.6%, n = 42), equitability (62.1%, n = 36), prompt attention (timeliness of care) (59.4%, n = 38), convenience (56.7%, n = 34), choice (selection of FCPS) (53.1%, n = 34), access to social support (51.7%, n = 30), and respect (50.6%, n = 56). Lower levels of agreement (<50%) were reported for other factors, including prompt attention (investigations) (38.7%, n = 24), choice (in-person vs telehealth) (31.3%, n = 20), and confidentiality (5.9%, n = 4).*** *In contrast, Australian respondents reported moderate to high agreement (≥50%) with only four responsiveness items: communication (90%, n = 36), prompt attention (timeliness of care) (66.7%, n = 28), autonomy (60%, n = 24), and equitability (60.5%, n = 23). The remaining items recorded lower levels of agreement (<50%), including prompt attention (investigations) (42.7%, n = 17), convenience (30%, n = 12), confidentiality (23.7%, n=9), access to social support (23.7%, n=9), choice (****selection of FCPS****) (18.8%, n=6), choice (****in-person vs telehealth****) (16.7% (n=5)), respect (7.5%, n=3)”*

**Minor comments:**
- Suggest using “reported changes” in the title

***Response:*** *“Reported” has been added to the title*
- Fig 1 axes should be number of respondents, not frequency.

***Response:*** *Frequency replaced with number of respondents*
- Throughout the manuscript, clarify that when analysing responses as a function of country, this is really a function of country + type of service (ED and PC) because of the degeneracy with which the survey was constructed. Maybe this combination could be redefined as “setting”.

***Response:*** *The abstract, methods (under data analysis), results and discussion sections have clearly stated that the analysis of survey responses and results presented by country actually reflect a function of both country and their FCPS context (UK [PC] and Australia [ED]).*

**Reviewer #3:** Thanks for your effort to cover the comments but in most cases, the answers and edits made did not cover the reviewer's comments.

***Response:*** *Thank you for your review. We note that Reviewer 1 acknowledged that the points raised have been addressed, stating: “The authors have comprehensively addressed the points raised in my first review. I believe currently there is only one major outstanding issue that should be addressed by the authors. I also include a few minor comments.” In the current revision, we have comprehensively addressed the single outstanding issue from Reviewer 1. Therefore, we are unclear about what additional changes Reviewer 3 is requesting, but we would be happy to consider them if we receive further information.*

**Reviewer #4:** Authors carefully responded to Reviewer 1's remarks by revising the title, abstract, introduction, and discussion for clearer definition of the scope of the study on first, perceived, and self-reported changes early in COVID-19 in First Contact Physiotherapy Services (FCPS). They acknowledged limitations such as recall bias, ambiguity in definitions, and sample representativeness. Technical terms such as "adaptations", "timing" were also clarified to prevent misinterpretation. Findings and tables were rewritten to ensure accuracy and unsubstantiated claims were removed and made more specific. Structural and grammatical suggestions for introduction and discussion were applied. Editorial requests were accommodated, such as ethics clarification, participant consent information, data availability via a DOI, and format changes according to PLOS ONE guidelines. Overall, the authors thoroughly revised with substantive changes that sufficiently resolved the reviewer's and editor's concerns and made the manuscript more transparent, consistent, and scientifically valid. Revisions reflect a strong effort to align the content with journal standards and reviewer expectations.

***Response:*** *Thank you for taking the time to review our paper and for your generous comments.*

**Reviewer #5:** The definitions of "rapidly implemented changes" and the vagueness of the survey questions could cause respondents to perceive them differently. Concerns around recall bias are further raised by the use of self-reported data. The findings are not well represented in the conclusion, and the authors ought to make a clearer contrast between observed changes in practice and official policy changes. The approach, a cross-sectional survey using physiotherapists' self-reported data, is suitable for documenting perceived changes. However, issues with recall bias and the imprecise definition of "first alterations" could compromise the validity of the results. Overall, even though the goal is pertinent, the method's drawbacks must be carefully considered. Resolving these *problems could improve the validity and significance of the study.*

***Response:*** *Thanks for reviewing our paper. While all these points were addressed in the previous revision (see our earlier responses to reviewers), a summary of those revisions is provided below:*

*The paper was revised all through to clearly state that the findings are based on providers perceived changes, and the title has also been updated accordingly.*

*The limitations section clearly highlighted and discussed the recall bias (paged 21 and 22).*

*In addition, the conclusion has been revised to more accurately reflect the study’s findings (reported changes).*

**Reviewer #6:** Thank you for the invitation to review the manuscript titled “Weathering the storm of COVID-19 pandemic: A cross-sectional survey of changes in first contact physiotherapy services in the UK and Australia” The manuscript is concise and scientifically written using recent literature
I have the following comments to offer in order to improve the quality of the study for use especially during pandemic period to enhance healthcare services in the two regions and globally.

1. Authors should give adequate explanation to changes in service delivery.

***Response:*** *Thank you for reviewing our paper. Changes in service delivery is now described in the introduction section (page 4) as below:*

*“For this study, “changes” refers to the transformations in the delivery of healthcare by FCPS to patients with MSK conditions during the COVID-19 pandemic [33]”.*

2. Conclusion in the abstract should highlight the effect on changes in service delivery in relation to future pandemic.

***Response:*** *Thank you for your suggestion the conclusion subsection in the abstract has highlighted the effect of changes in the context of managing future pandemic.*

3. What are the initial changes noticed in UK and Australia during the pandemic?

***Response:*** *The manuscript already highlighted changes in the UK and Australia, and the results presented initial changes in FCPS*

4. How did the authors ensure the participants were able to recall changes in their practice during the pandemic, was any instrument used to ascertain cognitive function?

***Response:*** *No cognitive function tests were required, as participants were practicing physiotherapists without cognitive impairments. Additionally, recall bias has been thoroughly addressed in the paper, including through survey piloting and discussion of limitations.*

5. What is the educational level of the participants?

***Response: Many thanks for this comment.*** *Educational level was presented in Table 2 under participant characteristics.*

6. The sample size is a bit low to make conclusion based on the findings.

***Response:*** *This is an exploratory study which used a non-probability sampling method, reporting perceived changes, hence the sampling is suitable for this type of research [35]. However, sample size has been acknowledged as one of the study limitations on page 22*

7. Explain the experiences and qualifications of the 12 participants drawn for the pilot test of the study instrument.

***Response:*** *Thank you for the suggestion. The experiences and qualifications of the 12 participants involved in the pilot testing of the study instrument have been included under the section describing the survey instrument (page 5) as below:*

*“The 12 participants hold master’s degrees, with 75% (n = 9) having more than four years of experience in FCPS, and 25% (n = 3) having three years of experience”*

8. In Table 1, the first column should have a heading like item or domain for clarity

***Response:*** *Thank you for your suggestion. The first column has been renamed as “Domains”.*

9. Recommendation for future study should be incorporated to address gaps in the existing study.

***Response:*** *Thank you very much for your suggestion. A recommendation for future study section has been added on the paper please see page 22 and below:*

*“Future research should investigate how changes in FCPS during the COVID-19 pandemic impacted MSK patients’ health outcomes and satisfaction. Additionally, further study is required to understand how telehealth can be effectively utilised to provide MSK care in FCPS especially within the ED. Evaluating both the effectiveness of readiness strategies, including telehealth interventions, as well as stakeholders’ experiences with these approaches, is essential to identify best practices for sustaining FCPS during crises. Qualitative methods, such as interviews and focus groups, could offer valuable insights into the experiences, challenges, and facilitators involved. It is also valuable to conduct an in-depth comparison of readiness and responsiveness across various healthcare systems and policy environments to better understand how structural and system level factors may impact FCPS service delivery. Additionally, a longitudinal approach to research can be employed to track how FCPS readiness and responsiveness evolves over time”*

10. Any conflict of interest resolved by the authors?

***Response:*** *The authors would like to declare that there was no conflict of interest. This declaration has been submitted via the journal’s submission system, in accordance with its requirements.*

**Editor:** Specifically, please consider addressing the complexity around country specific readiness and the impact of the divergences within the included primary literature.

***Response****: Thank you for this comment, which provides clear direction on how to proceed. Details regarding responsiveness and readiness across both countries, along with potential variations and complexities have been addressed and revised in the Abstract, Results and Discussion sections.*

While revising your submission, please upload your figure files to the Preflight Analysis and Conversion Engine (PACE) digital diagnostic tool, [https://pacev2.apexcovantage.com/](https://eur02.safelinks.protection.outlook.com/?url=https%3A%2F%2Ftrack.editorialmanager.com%2FCL0%2Fhttps%3A%252F%252Fpacev2.apexcovantage.com%252F%2F1%2F010f0197c50c6dfb-b70e5fe6-20bf-47f2-83e6-c2fbb15bc7c8-000000%2Fc3bLps33r6tOZTGDkK2lbOqzSRPx-O6M6UsoSDOSRTY%3D217&data=05%7C02%7Co.adeniji%40surrey.ac.uk%7Cbdffdaad4f7249e11c9a08ddb8772e01%7C6b902693107440aa9e21d89446a2ebb5%7C0%7C0%7C638869544778774536%7CUnknown%7CTWFpbGZsb3d8eyJFbXB0eU1hcGkiOnRydWUsIlYiOiIwLjAuMDAwMCIsIlAiOiJXaW4zMiIsIkFOIjoiTWFpbCIsIldUIjoyfQ%3D%3D%7C0%7C%7C%7C&sdata=DPpvJnWHFHBkDQh2eEiWBZrf4xcmctquMJ%2FykwUJIn8%3D&reserved=0). PACE helps ensure that figures meet PLOS requirements. To use PACE, you must first register as a user. Registration is free. Then, login and navigate to the UPLOAD tab, where you will find detailed instructions on how to use the tool. If you encounter any issues or have any questions when using PACE, please email PLOS at [figures@plos.org](mailto:figures@plos.org). Please note that Supporting Information files do not need this step.

***Response:*** *Please note that as requested, the PACE output for Figure 1 has now been included in the resubmission.*
