# Supplementary material for: Weathering the storm of COVID-19 pandemic: A cross-sectional survey of reported changes in first contact physiotherapy services in the UK and Australia
Source: PLoS One. 2026 Jan 21;21(1):e0340995. doi: 10.1371/journal.pone.0340995 (PMC12822978; doi:10.1371/journal.pone.0340995)
Supplement: Attachment — Submitted filename: Response to editor.docx [file pone.0340995.s007.docx]

Response to editor

Thanks to reviewers and editor for reviewing our revised paper.

**Editor**

The reviewers had no additional comments and confirmed that all previous suggestions had been fully addressed. The final version of the manuscript has now been resubmitted for your consideration.

Thank you for your time and continued support throughout the review process.
