# Supplementary material for: Weathering the storm of COVID-19 pandemic: A cross-sectional survey of reported changes in first contact physiotherapy services in the UK and Australia
Source: PLoS One. 2026 Jan 21;21(1):e0340995. doi: 10.1371/journal.pone.0340995 (PMC12822978; doi:10.1371/journal.pone.0340995)
Supplement: Attachment — Submitted filename: WEATHERING REVIEWER COMMENTS.docx [file pone.0340995.s004.docx]

Thank you for the invitation to review the manuscript titled “Weathering the storm of COVID-19 pandemic: A cross-sectional survey of changes in first contact physiotherapy services in the UK and Australia”

The manuscript is concise and scientifically written using recent literature

I have the following comments to offer in order to improve the quality of the study for use especially during pandemic period to enhance healthcare services in the two regions and globally

Reviewer: Adamu Ahmad Adamu PT PhD

Comments:

1. Authors should give adequate explanation to changes in service delivery
2. Conclusion in the abstract should highlight the effect on changes in service delivery in relation to future pandemic
3. What are the initial changes noticed in UK and Australia during the pandemic?
4. How did the authors ensured the participants were able to recall changes in their practice during the pandemic, was any instrument used to ascertain cognitive function?
5. What is the educational level of the participants?
6. The sample size is a bit low to make conclusion based on the findings
7. Explain the experiences and qualifications of the 12 participants drawn for the pilot test of the study instrument
8. In Table 1, the first column should have a heading like item or domain for clarity
9. Recommendation for future study should be incorporated to address gaps in the existing study
10. Any conflict of interest resolved by the authors?
